# Daily challenge-hindrance stress and work engagement in preschool teacher: the role of affect and mindfulness

**DOI:** 10.1186/s12889-024-20255-9

**Published:** 2024-10-11

**Authors:** Jinghui Zhang, Qing Zhang, Yingjie Wang, Bowen Xiao, Shuming Wang, Yige Xu, Yan Li

**Affiliations:** 1 Shanghai Institute of Early Childhood Education, Shanghai Normal University, 100 Guilin Road, Xuhui District, Shanghai, China; 2 School of Teacher Education, Huzhou University, Huzhou, China; 3 Psychology Department, Carleton University, Ottawa, Canada

**Keywords:** Preschool teacher, Stressors, Affect, Work engagement, Mindfulness

## Abstract

**Background:**

The engagement of preschool teachers in their work is pivotal for maintaining teaching quality, ensuring teacher well-being, and fostering children’s development. Despite its significance, there is limited knowledge regarding the daily fluctuations in work engagement and the underlying factors influencing it. This study, guided by the Job Demands-Resources model and Affect Event Theory, utilized an experience sampling methodology to investigate the impact of challenge and hindrance stressors on daily work engagement, as well as the mediating role of affect and the moderating effect of mindfulness.

**Methods:**

Utilizing an experience sampling method, this study collected data from 220 preschool teachers in Shanghai over five consecutive workdays, conducting surveys once daily. Data analysis was performed using multilevel linear models.

**Results:**

The results from multilevel regression indicated that: (1) daily challenge stressors were positively related to work engagement, (2) daily hindrance stressors were negatively related to work engagement, (3) daily positive affect mediated the relationship between challenge stressors and work engagement, (4) daily negative affect mediated the relationship between hindrance stressors and work engagement, and (5) daily mindfulness played a crucial moderating role by alleviating the adverse effects of hindrance stressors on daily negative affect.

**Conclusions:**

This study provides valuable insights into the daily experiences of preschool teachers and the factors that influence their work engagement. Understanding the impact of stressors, affect, and mindfulness on work engagement can inform the development of interventions and strategies to improve teacher well-being and work engagement.

## Background

Teacher work engagement, defined as teachers willingly dedicating their physical, cognitive, and emotional resources to teaching activities [1, 2], is vital for teaching quality, teacher well-being, and children’s development [3–5]. Engaged teachers show higher motivation, job satisfaction, and retention rates [3, 6]. Longitudinal studies link work engagement to positive emotions, enhanced self-efficacy, and improved mental health [7, 8]. However, preschool teachers face significant challenges—such as low wages, lack of benefits, suboptimal work environments, and limited career advancement opportunities—that threaten their work engagement [9–11]. Furthermore, work engagement is susceptible to changes and fluctuations in daily experiences [3, 12, 13]. While trait work engagement focuses on stable individual differences, state work engagement captures within-person fluctuations [13, 14]. Examining state work engagement is crucial for understanding daily variations and developing strategies to enhance engagement and satisfaction. This study aims to investigate the daily factors influencing preschool teachers’ work engagement and the underlying mechanisms, guided by the Job Demands-Resources (JD-R) model [15, 16] and Affective Events Theory [17].

The JD-R model posits that job demands influence work engagement [15, 16], categorizing them into challenge stressors and hindrance stressors. Challenge stressors are perceived as obstacles that can be overcome and have positive implications for personal growth, such as time constraints and task complexity [18]. Hindrance stressors are viewed as barriers hindering goal achievement and career development, like organizational politics and role ambiguity [19–22]. According to the JD-R model and Transactional Theory [23, 24], challenge stressors stimulate motivation, leading to higher work engagement, while hindrance stressors result in negative appraisals and lower engagement. Empirical studies support these theories, showing that challenge stressors are positively associated with work engagement, whereas hindrance stressors are negatively associated [25–28]. Understanding how these stressors affect preschool teachers is essential for promoting their work engagement.

Affective Events Theory [17] suggests that workplace events elicit affective responses that influence work behaviors. Affect is generally categorized as positive and negative [29, 30]. Challenge and hindrance stressors are evaluated by individuals to determine their significance and importance, which in turn affects their affect response [31]. Challenge stressors likely evoke positive affect—feelings of enthusiasm and alertness—which enhance work engagement [19, 26, 32]. In contrast, hindrance stressors may elicit negative affect—such as anger and anxiety—diminishing work engagement [26, 32]. The broaden-and-build theory [33–35] explains that positive affect broadens thought-action repertoires and builds personal resources, promoting engagement. Negative affect depletes resources and narrows focus, adversely affecting engagement. Studies confirm the mediating role of affect between stressors and work engagement [36–38]. Examining this mediation can elucidate how stressors impact teachers’ engagement.

The JD-R model highlights personal resources that mitigate negative effects of job demands [15]. Mindfulness, defined as intentional and nonjudgmental present-moment awareness [39], serves as a valuable personal resource. Viewed as a transient state [40], it includes acting with awareness, nonjudgmental acceptance, and present-moment attention [41]. In workplaces, mindfulness enhances well-being, job satisfaction, and work engagement while reducing stress [42–45]. The mindful coping model [46] posits that mindfulness enables reappraisal of stressful events, reducing negative affect. Empirical research shows that mindfulness moderates the relationship between stressors and emotional responses, aiding adaptation to challenges [47, 48]. Investigating mindfulness’s moderating role can offer insights into how preschool teachers manage hindrance stressors.

Despite preschool teachers’ critical role, there is a gap in understanding how daily stressors affect their work engagement. Past research focused on trait perspectives, overlooking daily fluctuations. Using ESM, this study captures the dynamic nature of work engagement. ESM allows for capturing dynamic processes, reducing recall bias, enhancing ecological validity, and examining within-person processes. This approach provides a more accurate representation of teachers’ experiences and minimizes the potential for retrospective bias inherent in traditional cross-sectional designs. This study aims to provide a more nuanced understanding of the factors influencing preschool teachers’ daily work engagement by examining the impact of daily challenge and hindrance stressors on work engagement, the mediating roles of positive and negative affect, and the moderating role of daily mindfulness. Based on theoretical frameworks and previous research, we propose:

*H1: Daily challenge stressors are positively related to preschool teachers’ daily work engagement.*

*H2: Daily hindrance stressors are negatively related to preschool teachers’ daily work engagement.*

*H3: Daily positive affect mediates the relationship between daily challenge stressors and work engagement.*

*H4: Daily negative affect mediates the relationship between daily hindrance stressors and work engagement.*

*H5: Daily mindfulness moderates the relationship between daily hindrance stressors and negative affect; specifically*,* the positive relationship is weaker for teachers with higher mindfulness levels.*

**Fig. 1:**
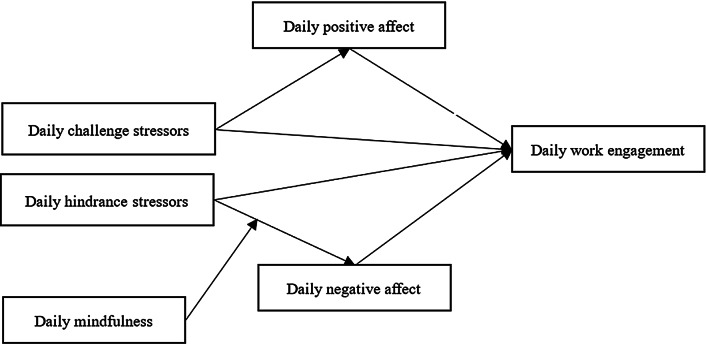
The hypothesized model

The conceptual model is shown in Fig. 1. By addressing these hypotheses, this study contributes a nuanced understanding of factors influencing preschool teachers’ daily work engagement, providing insights for interventions to enhance their well-being and effectiveness in early childhood education.

## Methods

### Participants

A total of 242 teachers from 11 kindergartens in Shanghai were surveyed in this study. Data were collected using Experience Sampling Methodology, and the survey lasted for 5 days. During this period, 196 teachers completed the survey for all 5 days, 24 teachers completed it for 4 days, 8 teachers for 3 days, 5 teachers for 2 days, and 9 teachers for 1 day. For the analysis, only participants who completed the survey 4 times or more were included, resulting in a final sample size of 220 participants. A total of 1076 individual-level data were collected. Among these 220 teachers, only one was male, while the remaining 219 were female. In terms of educational background, 58 teachers had a junior college education or below, 160 had a college education, and 2 had a graduate education or higher. The average teaching experience of the teachers was 3.08 years (*SD* = 1.67), and their average age was 32.35 years (*SD* = 7.70).

### Procedure

Before collecting data, the study received ethical approval from the institutional review board of Shanghai Normal University. Then, the researchers introduced the purpose of the research to the kindergarten teachers, and an electronic version of the “Informed Consent Form” was then distributed to each teacher through the Wenjuanxing platform to obtain their voluntary participation in the subsequent survey. Data collection was conducted online using the Wenjuanxing platform, consisting of two stages. In the first stage, a general questionnaire was administered to collect basic information such as gender, educational background, teaching experience, age, and email address from the teachers. The second stage involved the use of Experience Sampling Method (ESM) to collect data, which lasted for 5 consecutive days (weekdays). Each day, a survey was conducted once. For teachers who agreed to participate in the research, a WeChat group was established for each kindergarten. During the ESM data collection stage, at 3:30 PM each day, the researchers distributed the online survey questionnaire link for that day in the WeChat group. Teachers were required to complete and submit the questionnaire within one hour of receiving the message. The daily survey questionnaire was relatively short and could be completed within 5 min. If any teacher had not submitted the questionnaire by 4:30 PM, a reminder would be sent in the group. The questionnaire response permission would be closed at 5:30 PM. If any teacher still had not submitted the questionnaire by 5:30 PM, their data for that day would be treated as missing. After completing both stages of the survey, a “Teacher Personality Analysis and Improvement Suggestions Report” would be sent to each participating teacher’s provided email address as a token of appreciation, aiming to help teachers gain a better understanding of their teaching characteristics and support their professional development.

### Measures

#### Challenge-hindrance stressors

The Challenge and Hindrance stressors scale developed by Rodell and Judge [49] was used to measure teachers’ daily perceptions of challenge stressors and hindrance stressors. This scale consists of 16 items, with two dimensions: challenge stressors and hindrance stressors. Participants rated each item on a 5-point scale (1 = strongly disagree, 5 = strongly agree). The challenge stressors subscale includes eight items that assess work demands, time pressure, job responsibilities, and job complexity. Example items include “Today, my job has required me to use a number of complex or high-level skills”. The hindrance stressors subscale also includes eight items that measure bureaucratic procedures, role ambiguity, role conflict, and job frustrations. Example items include “Today, I have not fully understood what is expected of me”. This shortened version has been validated in the Chinese context, demonstrating good measurement properties [50]. To reduce the daily response burden on participants, this study also employed the shortened version of eight items to measure daily challenge stressors and hindrance stressors. The Cronbach’s alpha reliability coefficients for challenge stressors ranged from 0.663 to 0.808 (average 0.745), and for hindrance stressors, they ranged from 0.725 to 0.791 (average 0.760) across the five days of measurement.

#### Work engagement

In order to assess teachers’ daily perception of work engagement, the 3-item version of the Utrecht Work Engagement Scale (UWES) was utilized [51]. The UWES measures three dimensions: vigor, dedication, and absorption, with one representative item for each dimension. Participants rated each item on a 5-point scale ranging from 1 (strongly disagree) to 5 (strongly agree). The effectiveness and reliability of the UWES-3 as a measure of work engagement have been demonstrated in samples from five countries [51]. In this study, the Cronbach’s alpha coefficients for work engagement across the five measurement points ranged from 0.860 to 0.934, with an average of 0.908, indicating good internal consistency reliability. However, given that each dimension is measured by a single item, Cronbach’s alpha may not fully capture the reliability of the scale. Therefore, we also calculated the Intraclass Correlation Coefficient (ICC) to assess the reliability of the UWES-3 in our repeated measurements design. The ICC for the UWES-3 was 0.53, indicating moderate reliability. This value suggests that 53% of the variance in work engagement scores is attributable to between-person differences, while 47% reflects within-person fluctuations. It demonstrates that the UWES-3 captures both trait-like (between-person) and state-like (within-person) aspects of work engagement, which is crucial for our daily diary design. We chose the UWES-3 to balance measurement quality with participant burden, a common practice in experience sampling studies. Despite its brevity, the UWES-3 provides a reliable measure of overall work engagement, facilitating the investigation of daily fluctuations and their predictors in our study.

#### Affect

The Positive and Negative Affect Schedule (PANAS) scales developed by Watson et al. [30] were used to measure daily positive and negative affect. Qiu et al. [52] adapted the scales for use in China, resulting in a revised version with two dimensions: positive affect and negative affect. Each dimension consisted of nine adjective items that described various emotions, totaling 18 items. Examples of positive affect adjectives included “enthusiastic”, “interested”, and “excited”, while negative affect adjectives included “upset”, “nervous”, and “scared.” Participants rated each item on a 5-point Likert scale, ranging from 1 (almost never) to 5 (very strongly). To calculate scores, the ratings for the positive affect and negative affect items were separately summed, resulting in scores for positive affect and negative affect. Previous studies using experience sampling methods have demonstrated its reliability and validity for assessing daily emotional experiences in Chinese samples [53]. The Cronbach’s alpha reliability coefficients for positive affect ranged from 0.949 to 0.975 (average 0.966), and for negative affect, they ranged from 0.917 to 0.946 (average 0.937) across the five days of measurement.

#### Mindfulness

The Multidimensional State Mindfulness Questionnaire (MSMQ), developed by Blanke and Brose [41], was utilized in this study to measure daily mindfulness. Zhou et al. [54] adapted the questionnaire for the Chinese context. The questionnaire comprises 9 items, which are categorized into three dimensions: acting with awareness, present moment attention, and nonjudgement. Participants rated each item on a 7-point Likert scale, ranging from 1 (completely inconsistent) to 5 (completely consistent). Higher scores on the scale indicate higher levels of mindfulness in daily life. The Cronbach’s alpha coefficients for daily mindfulness in this study ranged from 0.674 to 0.752, with an average of 0.730 across the 5 measurement occasions.

### Statistical analysis

The data collected for this study was obtained using the experience sampling methodology and exhibit a nested structure, with multiple days of measurement nested within individuals. Specifically, we assessed daily hindrance stressors, challenge stressors, positive affect, negative affect, and work engagement at the within-person level (level 1). In contrast, teacher characteristics such as age, teaching experience, and education level were measured at the between-person level (level 2). Given this data structure, multilevel linear models (MLM) were employed for analysis [55].

Before testing the hypotheses, we examined the appropriateness of multilevel analyses by assessing the within- and between-individual variance in the variables. The results, presented in Table 1, revealed that a substantial proportion of the total variance in the variables (ranging from 29.16 to 54.71%) was accounted for by within-individual differences. This within-individual variance provided support for proceeding with MLM, as there was both within- and between-individual variance to be explained.

**Table 1:** Variance components of null models for day level variables

Variables	Fixed intercept	Between-person variance	Within-person variance	1 - ICC
Daily challenge stressors	3.599^***^	0.308	0.372	54.71%
Daily hindrance stressors	1.875^***^	0.324	0.256	44.14%
Daily Positive affect	3.361^***^	0.809	0.333	29.16%
Daily Negative affect	1.474^***^	0.298	0.171	36.46%
Daily Work engagement	3.959^***^	0.428	0.382	47.16%

*Note* The ICC is calculated by dividing between-person variance by the sum of the within-person and between-person variances. The value of the ICC indicates the percentage of between-person variance. The within-person variance (percentage) is calculated as 1 - ICC. ^***^p < .001

To control for potential confounding effects, teacher characteristics such as age, teaching experience, and education level were included as control variables in the models during hypothesis testing. Following the suggestion of Hofmann and Gavin [56] regarding variable centering, all level 1 variables were mean-centered for each participant to eliminate between-person variability. Additionally, all level 2 variables were grand-mean centered.

The hypothesis testing proceeded in three steps. First, multilevel analysis using the lme4 package in R was conducted to examine the impact of daily challenge stressors and daily hindrance stressors on daily work engagement. Second, the Mlmed was utilized to test for emotional mediation effects. Mlmed is specifically designed for conducting multilevel mediation and moderation analyses, allowing for the inclusion of multiple mediators and providing convenient confidence intervals for the mediation effects [57]. However, Mlmed does not account for the moderation effects of level 1 variables. Therefore, as a third step, multilevel linear models using the lme4 package in R were constructed to test the moderating effect of daily mindfulness. To visually represent the moderating effect more clearly, Johnson-Neyman plots were generated using the R package “interactions” [58].

## Results

### Descriptive analyses

Table 2 presents the means, standard deviations, and correlations for all studied variables. The correlation analysis revealed significant associations, both at the between-person and within-person levels, among challenge stressors, hindrance stressors, positive affect, negative affect, and work engagement.

**Table 2:** Mean, standard deviations, and correlations for the study variables

Variable	Mean	SD	1	2	3	4	5	6	7	8
1.DCS	3.60	0.62 (0.82)	1	0.296^***^	0.205^**^	0.176^**^	0.158^*^	0.127	0.109	0.166^*^
2.DHS	1.88	0.61 (0.76)	0.269^***^	1	− 0.268^***^	0.531^***^	− 0.421^***^	0.047	0.050	0.053
3.DPA	3.36	0.94 (1.07)	0.173^***^	− 0.202^***^	1	− 0.113	0.790^***^	− 0.012	− 0.064	− 0.050
4.DNA	1.47	0.58 (0.68)	0.135^***^	0.416^***^	− 0.109^***^	1	− 0.315^***^	− 0.002	− 0.027	− 0.014
5.DWE	3.96	0.71 (0.90)	0.178^***^	− 0.280^***^	0.683^***^	− 0.263^***^	1	0.018	− 0.042	− 0.116
6. Age	32.35	7.70						1	0.861^***^	0.011
7. Teaching	3.08	1.67							1	0.162^*^
8. Education	2.70	0.56								1

*Note* DCS = Daily challenge stressors, DHS = Daily hindrance stressors, DPA = Daily positive affect, DNA = Daily negative affect, DWE = Daily work engagement. SDs presented in parentheses are at the within-person level. Correlations above the diagonal are between-person correlations, while those below the diagonal are within-person correlations. Sample sizes for between-person correlations are 220 and within-person correlations are 1076. ^***^p < .001, ^**^p < .01, ^*^p < .05

### Hypothesis testing

Hypotheses 1 and 2 propose that daily challenge and hindrance stressors have opposite effects on daily work engagement. We conducted a multilevel regression analysis with the level 1 variables (i.e., daily challenge and hindrance stressors) predicting daily work engagement, while controlling for the level 2 variables (i.e., teachers’ age, education, and teaching experience). The results showed a positive relationship between daily challenge stressors and work engagement (*β* = 0.19, *p* < .001), and a negative relationship between daily hindrance stressors and work engagement (*β* = -0.10, *p* < .05), supporting Hypothesis 1 and 2 (see Model 1 in Table 3).

**Table 3:** Multilevel estimates for the daily stressors and affect on daily work engagement

Predictors	Model 1			Model 2		
	Estimate	SE	95% CI	Estimate	SE	95% CI
**Level 2 (person level)**						
Teach age	-1.87e-04	2.22e-3	(-0.005, 0.004)	-0.001	0.001	(-0.004, 0.001)
Teaching experience	-0.01	0.03	(-0.069, 0.049)	0.01	0.02	(-0.023, 0.044)
Teach education	-0.15	0.09	(-0.327, 0.024)	-0.16^*^	0.05	(-0.258, -0.058)
**Level 1 (day level)**						
Intercept	4.41^***^	0.27	(3.889, 4.926)	2.84^***^	0.22	(2.397, 3.277)
DCS	0.19^***^	0.05	(0.101, 0.283)	0.12^*^	0.04	(0.414, 0.198)
DHS	-0.10^*^	0.05	(-0.197, -0.009)	-0.04	0.04	(-0.121, 0.043)
DPA				0.40^***^	0.04	(0.316, 0.489)
DNA				-0.14^*^	0.05	(-0.237, -0.042)
**Residual variance**						
Level 1	0.287			0.167		
Level 2	0.447			0.117		

*Note* DCS = Daily challenge stressors, DHS = Daily hindrance stressors, DPA = Daily positive affect, DNA = Daily negative affect, DWE = Daily work engagement. ^***^p < .001, ^**^p < .01, ^*^p < .05

Hypotheses 3 and 4 proposed that daily challenge stressors and hindrance stressors, respectively, would influence daily work engagement through the mediating effects of daily positive affect and negative affect. The results of the mediation analysis supported these hypotheses. Specifically, daily challenge stressors were found to be positively related to daily positive affect (*β* = 0.146, *p* < .001) (see Model 1 in Table 4), while daily hindrance stressors were positively related to daily negative affect (*β* = 0.145, *p* < .01) (see Model 2 in Table 4). Additionally, daily positive affect was positively associated with daily work engagement (*β* = 0.40, *p* < .001), while daily negative affect was negatively associated with daily work engagement (*β* = -0.14, *p* < .05). When daily positive affect and negative affect were included as additional predictors in the model, the relationship between daily challenge stressors and daily work engagement remained significant (*β* = 0.12, *p* < .05), while the relationship between daily hindrance stressors and daily work engagement became non-significant (*β* = -0.04, *p* > .05). Refer to Model 2 in Table 3 for specific details. These findings indicate that daily positive affect acts as a mediator in the relationship between daily challenge stressors and work engagement, supporting Hypothesis 3. The mediating effect of daily positive affect was found to be significant through bootstrapping analysis (mediator effect = 0.059, *SE* = 0.017, *95% CI* = [0.028, 0.093]). Similarly, daily negative affect acts as a mediator in the relationship between daily hindrance stressors and work engagement, supporting Hypothesis 4. The mediating effect of daily negative affect was also found to be significant through bootstrapping analysis (mediator effect = -0.020, *SE* = 0.009, *95% CI* = [-0.040, -0.005]). In summary, the results provide support for Hypotheses 3 and 4, demonstrating that daily positive affect mediates the relationship between daily challenge stressors and work engagement, while daily negative affect mediates the relationship between daily hindrance stressors and work engagement. These findings highlight the importance of considering the role of affective experiences in understanding the impact of stressors on work engagement.

**Table 4:** Multilevel estimates for the daily stressors and mindfulness on daily positive affect and negative affect

Predictors	Model 1 (Daily positive affect)			Model 2 (Daily negative affect)			Model 3 (Daily negative affect)		
	*Estimate*	*SE*	95% CI	*Estimate*	*SE*	95% CI	*Estimate*	*SE*	95% CI
**Level 2 (person level)**									
Teach age	0.003	0.003	(-0.003, 0.008)	-0.003	0.002	(-0.006, 0.0004)	-0.002	0.002	(0.073, 0.272)
Teaching experience	-0.049	0.037	(-0.122, 0.024)	-0.003	0.021	(-0.044, 0.038)	0.013	0.023	(-0.004, 0.023)
Teach education	-0.102^*^	0.111	(-0.320, 0.116)	-0.17^*^	0.062	(-0.287, -0.042)	-0.147	0.069	(-2.128, 0.034)
**Level 1 (day level)**									
Intercept	2.941^***^	0.437	(2.078, 3.803)	0.916^**^	0.246	(0.430, 1.401)	1.875^***^	0.204	(0.134, 0.235)
DCS	0.146^***^	0.038	(0.070, 0.221)						
DHS				0.145^**^	0.037	(0.071, 0.219)	0.080^*^	0.036	(-0.581, -0.480)
DM							-0.237^***^	0.046	(-0.581, -0.480)
DM*DHS							-0.257^**^	0.094	(-2.727, 0.007)
**Residual variance**									
Level 1	0.299			0.141			0.132		
Level 2	0.679			0.206			0.303		

*Note* DCS = Daily challenge stressors, DHS = Daily hindrance stressors, DWE = Daily work engagement, DM = Daily mindfulness. ^***^p < .001, ^**^p < .01, ^*^p < .05

**Fig. 2:**
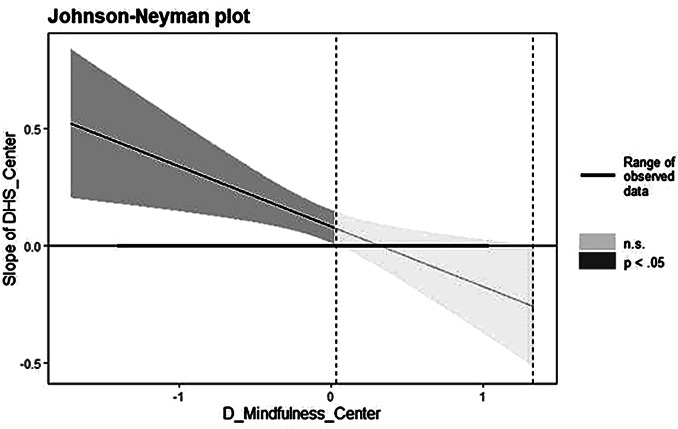
Johnson-Neyman plot demonstrates the moderating effect of daily mindfulness on the relationship between daily hindrance stressors and negative affect. *Note* The Y-axis represents the slope indicating the effect of daily hindrance stressors on negative affect at different levels of daily mindfulness. The X-axis represents the range of daily mindfulness values, which in this study falls within the range of (-1.40, 1.02). The bold black solid line represents the simple regression line, illustrating the relationship between daily hindrance stressors and daily negative affect across varying levels of mindfulness. The shaded area corresponds to the 95% confidence interval, indicating the region of statistical significance. The significant regions are depicted in black, while the nonsignificant region is shaded in grey. The vertical dashed line indicates the boundary of the significant region

Hypothesis 5 indicated that the relationship between daily hindrance stressors and negative affect would be moderated by daily mindfulness. The results, presented in Model 3 of Table 4, indicate a positive association between the interaction of daily hindrance stressors and mindfulness with negative affect (*β* = -0.257, *p* < .05). This suggests that daily mindfulness moderates the relationship between daily hindrance stressors and work engagement. To further explore how the impact of daily hindrance stressors on negative affect depends on the range of daily mindfulness, we employed the Johnson-Neyman technique to examine the moderating effects of daily mindfulness. Figure 2 illustrates the results. Within the range where daily mindfulness values is equal to or less than 0.03 (expressed in centered data, indicating 0.03 standard deviations above the mean), the confidence bands did not include zero. In this range, the positive relationship between daily hindrance stressors and negative affect was statistically significant, but its strength weakened as daily mindfulness increased. Moreover, when daily mindfulness values exceeded 0.03, as indicated by confidence bands containing zero, this positive relationship lost statistical significance. Therefore, the positive effect of daily hindrance stressors on negative affect was weakened and even became statistically insignificant when daily mindfulness reached higher levels. In summary, the findings support Hypothesis 5, demonstrating that daily mindfulness moderates the relationship between daily hindrance stressors and negative affect. The results suggest that higher levels of daily mindfulness can mitigate the impact of hindrance stressors on negative affect.

## Discussion

This study investigated the relationships between daily challenge and hindrance stressors, affect, mindfulness, and work engagement among preschool teachers. Our findings suggest significant associations between these variables, providing insights into the daily experiences of preschool teachers and factors related to their work engagement. In the following sections, we discuss these findings in detail, considering their theoretical implications and practical significance for early childhood education.

### Key findings

The current study supports hypothesis 1 and hypothesis 2 by revealing a positive association between daily challenge stressors and preschool teachers’ work engagement, and a negative association between daily hindrance stressors and work engagement. These results are consistent with previous empirical studies [25–28] that have found similar relationships. These findings align with the Job Demands-Resources (JD-R) model [8], which categorizes job demands into challenge and hindrance stressors. The Transactional Theory [23, 24] further suggests that individuals’ appraisal of stressors may be related to their level of work engagement.

The current study supports hypotheses 3 and 4, indicating that daily positive affect is associated with the relationship between daily challenge stressors and work engagement, while daily negative affect is associated with the relationship between daily hindrance stressors and work engagement. This is similar to previous research conducted by Rodell and Judge [49], Yang and Li [32], and Zhang et al. [36], who found associations between work stressors, emotional reactions, and work-related behavioral outcomes. These findings align with the affective events theory [9], transactional theory [23], and broaden-and-build theory [34].

The present study supports Hypothesis 5, indicating that daily mindfulness can moderate the relationship between daily hindrance stressors and daily negative affect. Higher levels of mindfulness are associated with a weaker relationship between daily hindrance stressors and daily negative affect. This finding both supports and extends the Job Demands-Resources model [8], which suggests that personal resources may be related to the effects of work demands on individuals. Consistent with the mindful coping model [46], our findings suggest that preschool teachers with higher levels of mindfulness may experience less negative affect in relation to daily hindrance stressors.

### Theoretical implications

First, this study contributes to the literature by examining work engagement among preschool teachers, an understudied yet crucial group in the education system. This focus enhances our understanding of work engagement in specific occupational contexts. Second, the use of experience sampling methodology provides a dynamic perspective on work engagement, capturing daily fluctuations and real-time experiences. This approach enriches our understanding of the day-to-day factors influencing work engagement, offering a more nuanced view than traditional cross-sectional studies. Third, our findings integrate and extend the Job Demands-Resources model and Affect Event Theory. By demonstrating the mediating role of daily affect in the relationship between stressors and work engagement, and the moderating role of mindfulness, we provide a more comprehensive framework for understanding the complex interplay of these factors. This integration offers new insights into the dynamic nature of work engagement and the role of personal resources like mindfulness in mitigating the effects of work stressors.

### Practical implications

For preschool teachers, recognizing daily fluctuations in stress, emotions, and work engagement is essential. Differentiating between challenge and hindrance stressors can inform strategies to maintain positive emotional experiences when facing challenges and seek support or utilize mindfulness techniques to mitigate negative impacts from hindrance stressors. For preschool administrators, the findings emphasize the importance of balancing task allocation to favor challenging over hindering demands. Implementing mindfulness training programs can enhance teachers’ resilience, while regular monitoring of work engagement, stressors, and emotional experiences through daily tracking tools can facilitate timely and targeted interventions. For policymakers, the study highlights two primary areas for action. First, supporting the implementation of mindfulness training programs for preschool teachers can improve their ability to manage work-related stress and enhance psychological well-being. Second, optimizing the work environment by reducing hindrance stressors and increasing challenge stressors—such as improving compensation, enhancing work conditions, and providing career advancement opportunities—can foster a more conducive environment for teacher engagement and development in early childhood education settings.

### Limitations and future directions

Despite the significant findings, this study is not without limitations, which provide directions for future research.

First, the most significant limitation of this study is the extreme gender imbalance in our sample, with only one male participant among 220 female teachers. This severe underrepresentation of male teachers significantly limits the generalizability of our findings. Our results should be interpreted as primarily applicable to female preschool teachers, and we cannot make any inferences about male teachers based on this study. This limitation reflects the broader gender imbalance in early childhood education but also highlights a critical area for future research. Subsequent studies should actively seek to include a more substantial proportion of male teachers to explore potential gender differences in the relationships we’ve examined.

Second, the study’s cultural context is limited to Shanghai, China. Cultural factors might have influenced the results, potentially limiting their generalizability to other contexts. Future research should consider replicating this study in diverse cultural settings to examine the universality of these findings and identify any culture-specific patterns in the relationships between stressors, mindfulness, affect, and work engagement among preschool teachers.

Third, while the instruments used in this study have been previously validated in Chinese contexts, a specific sample validation was not conducted for this study. This limitation extends to all measures used, including the ultra-short UWES-3 for work engagement. Future studies should consider conducting sample-specific validations to ensure the cultural and occupational appropriateness of these measures for Chinese preschool teachers. Additionally, the use of the UWES-3, while practical for reducing participant burden in experience sampling studies, may not capture the full complexity of the work engagement construct. Future research could benefit from using more comprehensive measures of work engagement, perhaps alternating between short and long forms across measurement occasions to balance measurement depth with participant burden. The wording of the UWES-3 items, directly adopted from Schaufeli et al. [51], may also need adaptation to better reflect the unique aspects of preschool teachers’ work engagement.

Fourth, this study relied solely on self-report measures, which may be subject to social desirability and recall biases. To address this limitation, future research could incorporate objective measures (e.g., observational data, peer-reports) to validate and complement self-report data. This multi-method approach would provide a more comprehensive and robust understanding of the phenomena under study.

Future research should address these limitations to provide a more comprehensive understanding of the relationships between daily stressors, mindfulness, affect, and work engagement among preschool teachers. This will further contribute to the development of effective stress management strategies and interventions in the field of early childhood education.

## Conclusion

This research highlights the contrasting effects of daily stressors on work engagement, with challenge stressors positively associated and hindrance stressors negatively associated. Moreover, this study sheds light on the mediating roles of daily positive and negative affect in these relationships. Additionally, it highlights the protective role of daily mindfulness in mitigating the negative impact of hindrance stressors. These findings provide a nuanced understanding of the mechanisms driving preschool teachers’ work engagement and emphasize the importance of mindfulness practices. Therefore, this study makes a significant contribution to the literature on occupational stress and work engagement in early childhood education, offering actionable insights for improving teacher well-being and productivity.

## Data Availability

The datasets used or analyzed during the current study are available from the corresponding author upon reasonable request.
